# The relationship between mean platelet volume and metabolic syndrome in patients with type 2 diabetes mellitus: A retrospective study

**DOI:** 10.1097/MD.0000000000025303

**Published:** 2021-04-02

**Authors:** Qinpei Ding, Fangwei Wang, Xintong Guo, Min Liang

**Affiliations:** a Department of Endocrinology; b Department of Respiratory Medicine, the First Affiliated Hospital of Guangxi Medical University, Nanning, China.

**Keywords:** mean platelet volume, metabolic syndrome, type 2 diabetes mellitus

## Abstract

This study aimed to investigate the association between mean platelet volume (MPV) and metabolic syndrome (MetS) in patients with type 2 diabetes mellitus (T2DM). Data for 1240 patients with T2DM admitted to the Department of Endocrinology at the First Affiliated Hospital of Guangxi Medical University between January 1, 2017 and June 1, 2020 were collected retrospectively via electronic medical records, including demographic information, complete blood count, lipid profile, and glucose metabolism indexes. MetS was defined according to the Chinese Diabetes Society. Among the 1240 patients enrolled, 873 (70.40%) had MetS. MPV was significantly higher in patients with MetS (*P* < .001). For individual MetS components, MPV was significantly higher in the presence of abdominal obesity (*P* = .013) and hypertriglyceridemia (*P* = .026), but did not differ in the presence of elevated blood pressure (*P* = .330) or low high-density lipoprotein cholesterol (*P* = .790). Moreover, MPV was independently associated with MetS after adjustment for sex, smoking, alcohol drinking, white blood cell count, fasting C-peptide, and body mass index (odds ratio 1.174, 95% confidence interval 1.059–1.302). The odds ratio for MetS in the highest tertile, compared with the lowest MPV tertile, was 1.724 (95% confidence interval 1.199–2.479, *P* for trend = .003) after multiple adjustment. In stratified analyses, the positive correlation of MPV with MetS was significant only in patients who were older, male, or overweight, or who had poor glycemic control. In conclusion, high MPV was positively associated with the presence of MetS in patients with T2DM, particularly older, male, or overweight patients, or those with poor glycemic control.

## 1 Introduction

Platelets play a crucial role in the development of atherothrombosis, a major contributor to cardiovascular events.^[1]^ Platelet activation and aggregation potential, the essential components of atherosclerosis, are central processes in the pathophysiology of cardiovascular diseases. Larger platelets are enzymatically and metabolically more active and have greater prothrombotic potential than smaller ones.^[2]^ Mean platelet volume (MPV) is a widely used marker of platelet size and activity, which is easily and inexpensively measured. Elevated MPV is associated with accelerated thrombopoiesis and an increased risk of cardiovascular diseases.^[3–5]^

Metabolic disorders such as dyslipidemia, obesity, and elevated blood pressure and glucose are risk factors for cardiovascular diseases.^[6]^ The presence of these metabolic disorders is responsible for the prothrombotic tendency, at least in part because of platelet hyperactivation.^[7]^ Numerous studies have strongly correlated these clinical features with platelet activation, as measured by MPV.^[8–15]^ For example, a retrospective analysis of 13,021 patients has shown a significantly higher MPV in patients with diabetes (8.2 vs 8.06 fL), and a significant correlation between MPV and glucose.^[10]^ The authors have concluded that MPV is strongly and independently associated with the presence and severity of diabetes. Another large sample study of 80,545 patients without cardiovascular diseases and hypertension has shown that elevated MPV is associated with prehypertension.^[16]^ Although a causative relationship between MPV and these metabolic disorders could not be addressed, these findings suggest that elevated MPV levels may link metabolic disorders and cardiovascular diseases.

Metabolic syndrome (MetS) is a group of cardiometabolic disorders, including central obesity, dyslipidemia, elevated blood pressure, and hyperglycemia.^[17,18]^ A relationship may potentially exist between MPV and MetS. Additionally, insulin resistance plays a central role in the pathogenesis of MetS,^[19]^ and insulin resistance is associated with increased platelet activity.^[7,20]^ To date, however, few studies have investigated the association between MPV and MetS, and the results are controversial.^[4,10,21–24]^ Further studies are warranted to validate a potential association. This retrospective study aimed to evaluate the relationship between MPV and MetS in patients with type 2 diabetes mellitus (T2DM).

## 2 Materials and methods

### 2.1 Research participants

This was a retrospective case–control study. Inpatients admitted to the Department of Endocrinology at the First Affiliated Hospital of Guangxi Medical University between January 1, 2017 and June 1, 2020 were reviewed via electronic medical records. The inclusion criteria consisted of the following:

(1) diagnosis with T2DM (on the basis of the diagnosis criteria of the World Health Organization in its published report of 1999^[25]^) without limitation of the duration of T2DM and the medications patients took; and
(2) aged ≥18 years.

The exclusion criteria included the following:

(1) hematological diseases;
(2) severe inflammatory or infectious diseases;
(3) acute coronary syndrome or severe heart failure (New York Heart Association class III to IV)^[26]^;
(4) hepatic or renal dysfunction (alanine aminotransferase and aspartate aminotransferase above 2 times the upper limit of normal, and creatinine > 2.5 mg/dL, respectively);
(5) malignancy or other critical diseases;
(6) lactation or pregnancy in women; and
(7) lack of necessary laboratory or physical examination data.

### 2.2 Data collection

For the included patients, clinical data were extracted from the electronic medical records, including age, sex, smoking (yes/no), alcohol drinking (yes/no), duration of T2DM, height, weight, waist circumference, hypertension (yes/no), systolic blood pressure (SBP), diastolic blood pressure (DBP), complete blood count, lipid profile, and glucose metabolism indexes. Blood samples were taken from a peripheral vein after an 8 to 12 hours overnight fast. Complete blood counts were measured with an automatic analyzer (Hitachi P800). Serum lipids and fasting plasma glucose were measured with enzymatic assays with an autoanalyzer (Beckman-Coulter LH780). HbA1c was measured with an automatic analyzer (TOSOH HLC-723G8). In our hospital, blood samples are routinely analyzed 1 to 3 hours after venipuncture.

Body mass index (BMI) was calculated as the weight in kilograms divided by the square of the height in meters. The insulin resistance index (homeostasis model assessment of insulin resistance [HOMA-IR]) was calculated with the updated homeostasis model assessment method (http://www.dtu.ox.ac.uk/) in HOMA2 Calculator (version2.2.3) software.^[27]^ Fasting plasma glucose and fasting C-peptide were used to calculate HOMA-IR. In patients with repeated hospitalization, the first medical record was used.

### 2.3 Definitions

MetS was defined according to the Chinese criteria^[28]^ as presentation of 3 or more of the following:

(1) abdominal obesity: waist circumference ≥90 cm for men or ≥85 cm for women;
(2) elevated blood pressure: SBP ≥ 130 mm Hg or DBP ≥ 85 mm Hg, or current use of antihypertensive medications;
(3) hypertriglyceridemia: triglyceride ≥ 1.7 mmol/L;
(4) low high-density lipoprotein cholesterol (HDL-C): HDL-C < 1.04 mmol/L; and
(5) hyperglycemia: fasting plasma glucose ≥ 6.1 mmol/L, or postprandial 2 hours plasma glucose ≥ 7.8 mmol/L, or previously diagnosed diabetes.

All patients had T2DM and, by definition, met the criteria for hyperglycemia. Overweight and obesity were defined as 24 ≤ BMI < 28 kg/m^2^ and BMI ≥ 28 kg/m^2^, respectively.^[29]^

### 2.4 Ethics statement

The study was conducted in accordance with the principles of Declaration of Helsinki, and was reviewed and approved by the Medical Ethics Committee of the First Affiliated Hospital of Guangxi Medical University [2020 (KY-E-096)]. Informed consent was exempted because of the nature of the retrospective research.

### 2.5 Statistical analysis

Statistical analyses were performed in SPSS software (version 17.0). The normality of the distribution of continuous variables was assessed with the Kolmogorov–Smirnov test. Continuous variables with a normal distribution are presented as mean ± SD and were analyzed by independent-sample *t* tests or one-way analysis of variance, whereas data with non-normal distributions are expressed as median (and interquartile range) and were analyzed with Mann–Whitney *U* tests. Categorical variables are given as frequency with percentage and were compared with *χ*^2^ tests. Associations between MPV and other variables were examined with Pearson or Spearman correlation coefficients. A logistic regression model was used to assess the odds ratios (ORs) and 95% confidence intervals (CIs) of having MetS, with adjustment for potential confounders. Tests of linear trends across increasing tertile of MPV were performed by assigning a grade (1, 2, or 3) to the corresponding tertile and treating it as a continuous variable. A two-sided *P* value < .05 was considered statistically significant.

## 3 Results

**Figure 1:**
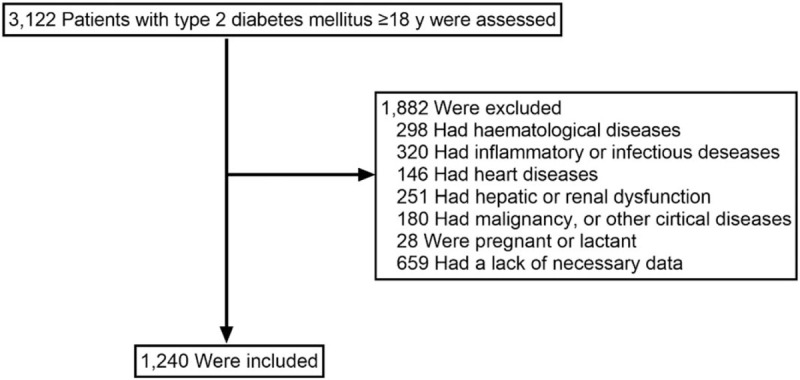
Flow diagram of patients excluded and included in the study.

A total of 3122 patients were screened; of those patients, 1240 were enrolled. A flow diagram of the excluded and included patients is provided in Figure 1. Among the 1240 patients, 873 (70.40%) had MetS. The mean age of the study population was 53 years (range 19–90 years), and 791 (63.79%) were male. As expected, patients with MetS had higher BMI, waist circumference, SBP, DBP, serum triglycerides, numbers of MetS components, HOMA-IR, and lower HDL-C than patients without MetS (all *P* < .05, Table 1). Additionally, the sex distribution, smoking proportion, alcohol drinking proportion, white blood cell count, and fasting C-peptide significantly differed between the patients with and without MetS (all *P* < .05, Table 1). However, no significant between-group difference was observed for age, duration of T2DM, red blood cell count, platelet count, plateletcrit, platelet distribution width, total cholesterol, low-density lipoprotein cholesterol, HbA1c, or fasting plasma glucose (all *P* > .05, Table 1). Of note, as reported in Table 1, patients with MetS had significantly higher MPV than patients without MetS (difference 0.53, 95% confidence interval [CI] 0.34–0.72, *P* < .001). Furthermore, patients in higher MPV tertile were more likely to be MetS (60.9% in tertile 1, 70.4% in tertile 2, 79.0% in tertile 3, *P* < .001).

**Table 1:** Demographic and clinical characteristics of the patients.

Variables	Without MetS	With MetS	*P*
Age (yr)	53.83 ± 12.46	53.36 ± 13.22	.561
Male sex	221 (58.5)	588 (66.4)	.007
Diabetic duration (yr)	3 (1, 10)	4 (1, 10)	.144
Smoking, yes	94 (24.9)	307 (34.6)	.001
Alcohol drinking, yes	84 (22.2)	271 (30.6)	.003
BMI (kg/m^2^)	22.21 ± 3.28	26.10 ± 3.98	<.001
Waist circumference (cm)	81.32 ± 9.05	92.93 ± 10.57	<.001
Hypertension, yes	91 (24.1)	533 (60.1)	<.001
SBP (mm Hg)	126.82 ± 20.20	137.63 ± 21.40	<.001
DBP (mm Hg)	75.40 ± 11.16	82.62 ± 12.94	<.001
White blood cell count (×10^9^/L)	6.93 ± 1.91	7.38 ± 2.10	<.001
Red blood cell count (×10^12^/L)	4.64 ± 0.66	4.72 ± 0.75	.065
Platelet count (×10^9^/L)	244.50 ± 72.84	244.46 ± 68.75	.993
Plateletcrit	0.22 ± 0.06	0.22 ± 0.05	.471
Platelet distribution width	0.15 ± 0.03	0.15 ± 0.02	.278
MPV (fL)	8.81 ± 1.60	9.34 ± 1.57	<.001
Total cholesterol (mmol/L)	4.89 ± 1.32	4.88 ± 1.42	.902
Triglyceride (mmol/L)	1.16 ± 0.56	2.21 ± 1.47	<.001
HDL-C (mmol/L)	1.22 ± 0.34	0.97 ± 0.27	<.001
LDL-C (mmol/L)	3.03 ± 1.10	2.91 ± 1.05	.060
HbA1c	9.59 ± 2.79	9.26 ± 2.43	.057
Fasting plasma glucose (mmol/L)	7.17 ± 3.13	7.45 ± 2.90	.126
Fasting C-peptide (ng/mL)	1.22 (0.51, 2.18)	2.09 (1.10, 2.96)	<.001
Number of components of MetS	1.66 ± 0.49	3.65 ± 0.89	<.001
HOMA-IR	1.15 (0.50, 2.00)	1.90 (1, 3)	<.001

BMI = body mass index, DBP = diastolic blood pressure, HDL-C = high-density lipoprotein cholesterol, HOMA-IR = homeostasis model assessment of insulin resistance, LDL-C = low-density lipoprotein cholesterol, MetS = metabolic syndrome, MPV = mean platelet volume, SBP = systolic blood pressure.

**Figure 2:**
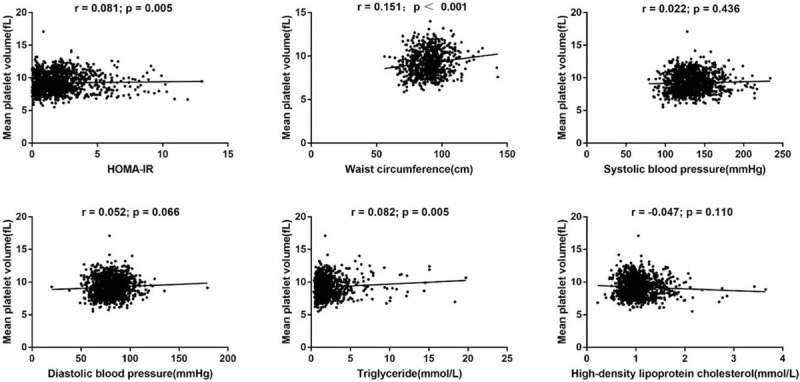
Correlation between mean platelet volume and metabolic parameters in patients with type 2 diabetes mellitus. HOMA-IR = homeostasis model assessment of insulin resistance.

Table 2 reports the mean MPV levels in patients with or without individual components of MetS except hyperglycemia. MPV was significantly higher in patients with abdominal obesity (*P* = .013), and hypertriglyceridemia (*P* = .026), but did not significantly differ for those with elevated blood pressure (*P* = .33) or low HDL-C (*P* = .79). The mean (SD) MPV levels (in fL) increased with the number of MetS components: 8.88 (1.62) if 1 or 2 components were present, 9.16 (1.63) if 3 components were present, 9.45 (1.55) if 4 components were present, and 9.51 (1.41) if 5 components were present (*P* < .001). As shown in Figure 2, MPV was significantly correlated with HOMA-IR, waist circumference, and serum triglycerides, whereas no significant associations were found with SBP, DBP, and HDL-C. Moreover, a significant correlation was observed between MPV and MetS (*r* = 0.164, *P* < .001) and between MPV and the number of components of MetS (*r* = 0.141, *P* < .001).

**Table 2:** Mean platelet volume levels (fL) according to the presence or absence of individual components of metabolic syndrome except hyperglycemia.

	Absent	Present	*P*
Abdominal obesity	8.99 ± 1.56	9.27 ± 1.55	.013
Elevated blood pressure	9.09 ± 1.61	9.19 ± 1.60	.330
Low HDL-C	9.15 ± 1.50	9.17 ± 1.68	.790
Hypertriglyceridemia	9.08 ± 1.66	9.29 ± 1.52	.026

HDL-C = high-density lipoprotein cholesterol.

**Figure 3:**
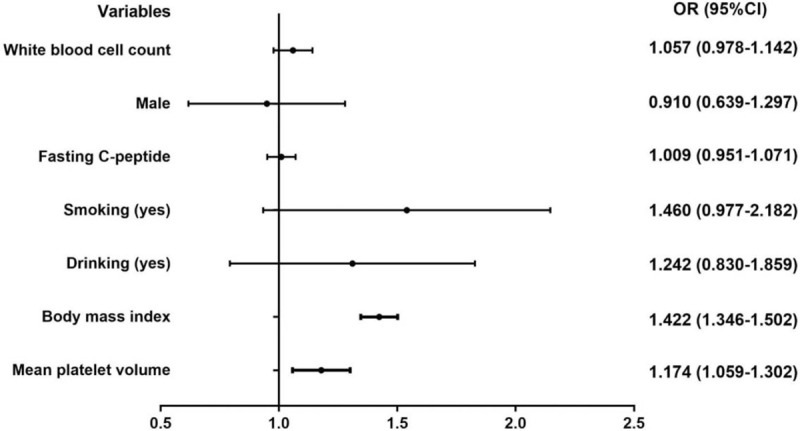
Logistic regression analysis of metabolic syndrome risk factors in patients with type 2 diabetes mellitus.

To analyze the association between MPV and MetS, we performed binary logistic regression analysis with MetS as a dependent variable. The significantly different variables in Table 1, except waist circumference, hypertension, SBP, DBP, triglycerides, HDL-C, number of components of MetS, and HOMA-IR, were taken as covariates. We excluded waist circumference, hypertension, SBP, DBP, triglycerides, and HDL-C as covariates, because they are components of MetS. Similarly, the number of components of MetS was also excluded. HOMA-IR was calculated on the basis of fasting C-peptide and, therefore, was also excluded to avoid multicollinearity. MPV was shown to be independently associated with MetS after adjustment for sex, smoking, alcohol drinking, white blood cell count, fasting C-peptide, and BMI (odds ratio [OR] 1.174, 95% CI 1.059–1.302, Fig. 3). After tertile classification of MPV, the risk of having MetS increased progressively across the lowest to the highest tertiles of MPV, with ORs of 1.559 (95% CI 1.164–2.089), and 2.500 (95% CI 1.816–3.442), respectively (*P* for trend < .001), after adjustment for sex, smoking, alcohol drinking, and white blood cell count (Model 1, Table 3). After further adjustment for fasting C-peptide, the ORs did not substantially change (Model 2). After additional control for BMI, the ORs were mildly attenuated (Model 3). In stratified analyses, the positive association remained only in patients who were older than 60 years or, male, and who had poor glycemic control (HbA1c ≥ 9%) or were overweight (24 ≤ BMI < 28 kg/m^2^) (Table 3).

**Table 3:** Adjusted ORs (95% CIs) of having metabolic syndrome according to tertiles of mean platelet volume.

	Mean platelet volume tertile			
	T1	T2	T3	*P* for trend
Model 1	1	1.559 (1.164–2.089)	2.500 (1.816–3.442)	<.001
Model 2	1	1.558 (1.153–2.104)	2.360 (1.699–3.277)	<.001
Model 3	1	1.341 (0.953–1.887)	1.724 (1.199–2.479)	.003
Age (Model 3)				
<45 yr (*n* = 351)	1	0.846 (0.423–1.694)	1.426 (0.680–2.992)	.326
45–60 yr (*n* = 496)	1	1.334 (0.738–2.410)	1.839 (0.963–3.511)	.063
≥60 yr (*n* = 393)	1	1.975 (1.122–3.475)	1.923 (1.083–3.416)	.020
Sex (Model 3)				
Male (*n* = 791)	1	1.240 (0.787–1.955)	1.674 (1.022–2.741)	.041
Female (*n* = 449)	1	1.369 (0.799–2.345)	1.732 (0.986–3.010)	.053
HbA1c (Model 3)				
<7% (*n* = 299)	1	1.540 (0.693–3.422)	1.510 (0.654–3.490)	.317
7%–9% (*n* = 374)	1	0.970 (0.469–2.006)	1.313 (0.616–2.800)	.486
≥9% (*n* = 567)	1	1.547 (0.945–2.531)	2.362 (1.374–4.061)	.002
BMI (Model 2)				
<24 kg/m^2^ (*n* = 531)	1	1.235 (0.810–1.881)	1.458 (0.931–2.283)	.094
24–28 kg/m^2^ (*n* = 476)	1	1.314 (0.718–2.402)	2.998 (1.428–6.294)	.004
≥28 kg/m^2^ (*n* = 233)	1	2.020 (0.497–8.208)	2.443 (0.592–10.077)	.222

BMI = body mass index, CI = confidence interval, OR = odds ratio. Model 1: adjusted for sex, smoking (yes/no), alcohol drinking (yes/no), and white blood cell count. Model 2: further adjusted for fasting C-peptide. Model 3: further adjusted for BMI.

## 4 Discussion

In the present study, we found that higher MPV was associated with an increased risk of MetS in patients with T2DM, particularly among those who were older, male, or overweight, or who had poor glycemic control. To our knowledge, this is the first study investigating the complex relationship between MPV and MetS in the setting of T2DM.

The positive association between MPV and MetS in our study is consistent with findings from several previous reports.^[4,20,23,30]^ For example, a study of 345 patients by Tavil et al^[23]^ has reported higher MPV values in patients with MetS referred for coronary angiography than in those without MetS, and that MetS is independently associated with MPV. Another study on 200 patients with MetS and 100 age- and sex-matched control subjects by Farah and Khamisy-Farah^[30]^ has shown a positive correlation between MPV and the presence and severity of MetS. Larger platelets, as measured by MPV, contain more denser granules and adhesion receptors, produce more serotonin and thromboglobulin, and show greater prothrombotic potential than smaller ones.^[2,31]^ The contribution of MetS to atherothrombosis has been noted for decades.^[32]^ Thus, the positive association between MPV and MetS appears credible, thereby suggesting that the association between MetS and cardiovascular events may occur to some extent through elevated platelet activity, as measured by MPV. The mechanism underlying higher MPV in MetS patients is incompletely understood and may be explained as follows. A feature of MetS is increasing adipose tissue, which secretes various adipokines and cytokines, including leptin, adiponectin, interleukin, and nitric oxide; these factors cause megakaryocytes to produce larger platelets.^[5,33]^ Additionally, vascular endothelial cells are severely damaged in patients with MetS, and endothelial dysfunction might also trigger the same mechanisms.^[5,34]^ Insulin resistance is a crucial mechanism underlying MetS. We found that MPV was positively correlated with insulin resistance, as measured by HOMA-IR, in agreement with the results of 2 previous studies.^[35,36]^ Hence, insulin resistance might plausibly link MPV and MetS.

Despite the above findings, several studies have reported an inverse association between MPV and MetS, only in women but not men.^[21,24,37]^ The inverse association among these studies was explained as follows: the platelet mass (platelet count × MPV) remained constant, and the platelet count was positively associated with MetS. Hence, the inverse association between MPV and MetS was subordinate to the positive association of platelet count with MetS. However, our study indicated that the platelet count in patients with MetS did not differ from that in patients without MetS. Furthermore, all patients in our study had T2DM, and a lack of an inverse relationship between MPV and platelet count has been reported in patients with T2DM (increased MPV and platelet count together).^[2,5]^ The differences according to sex were explained by different lifestyles (i.e., lower smoking and alcohol drinking rates in women than men), and a higher fat percentage in women than in men with the same BMI. In the present study, however, a positive association was observed between MPV and MetS in men but not women after adjustment for potential confounding factors, such as smoking and alcohol drinking. Thus, the potential sex effects of MPV on MetS must be elucidated further. In addition, some studies did not find that having MetS was associated with a significant difference in MPV values.^[10,22,38,39]^ These differences across studies may be explained in part by several factors such as the selection criteria (i.e., general population or diabetic patients), diagnostic criteria for MetS, race, and age (i.e., pre-pubertal children or older adults), thus suggesting that the association between MPV and MetS deserves further scrutiny.

In stratified analyses, we also found that MPV was associated with MetS in patients who were older or overweight, or who had poor glycemic control. MPV has been suggested to vary according to age,^[2]^ and to increase with aging.^[40]^ Older individuals usually show deteriorated cardiovascular homeostasis and severe metabolic disturbances, followed by increased platelet activity.^[5,41]^ MPV has consistently been associated with obesity.^[5]^ Weight loss in obese patients is also associated with a decrease in MPV.^[9]^ In the present study, we also found that MPV was positively associated with waist circumference, an indicator of obesity. Of note, we found that the association between MPV and MetS was significant only in overweight (24 ≤ BMI < 28 kg/m^2^) but not in obese (BMI ≥ 28 kg/m^2^) patients. This result is consistent with findings from a previous study of 920 patients (BMI ≥ 30 kg/m^2^) by Kutlucan et al, in which MPV did not differ between patients with versus without MetS.^[38]^ The authors offered some explanations that might also account for our results. When the BMI was sufficiently high, the effect of BMI increment and association of MetS components reduced to an insignificant level, although the BMI remained significantly different.^[38]^ This explanation appears to be credible and indirectly supports the close relationship between MPV and obesity. In our study, MPV was positively associated with MetS only in patients with poor glycemic control. Hyperglycemia leads to increased plasma osmotic pressure, thus resulting in osmotic swelling of platelet and elevated MPV.^[10]^ A previous study^[8]^ has reported decreased MPV during a 3-month follow-up in patients with diabetes who achieved improved glycemic control. The stronger association of MPV with MetS among patients with poor glycemic control suggests that good glycemic control may be helpful in decreasing platelet activity, thereby decreasing the incidence of cardiovascular events.

This study appears to be the first to investigate the complex relationship between MPV and MetS in the setting of T2DM. However, the lack of prior publications on this topic might be due to a lack of clinical significance. More studies on this topic should be performed in the future. Our study also has several limitations. First, a causal relationship between MPV and MetS could not be identified because of the case–control design. Second, the times between blood sample collection and analysis of the blood count (usually within 3 hours) may have differed among patients. Platelet volume increases over time in EDTA, the anticoagulant most extensively used in laboratory practice.^[2]^ However, platelet swelling in EDTA occurs mostly within 1 hour after venipuncture, and little change occurs after 2 hours.^[4]^ Hence, platelet swelling in EDTA should not have impaired the robustness of our findings. Third, all patients in our study were inpatients with T2DM, and those with severe complications were excluded, thus resulting in limited generalizability of our findings. Additionally, some potential confounding factors such as the use of medications (i.e., aspirin, clopidogrel, oral hypoglycemic agents or insulin, or oral contraceptives),^[42]^ coexisting cardiovascular diseases,^[5]^ diabetic complications, the menstruation status of women, and physical activity^[43]^ were not considered.

## 5 Conclusions

In summary, MPV was independently associated with MetS in patients with T2DM. The association of MPV with MetS was stronger in patients who were overweight, male, or older, or had poor glycemic control. Our findings suggest that in patients with T2DM, MPV may be another characteristic of MetS and may potentially be used as a surrogate indicator for MetS in clinical practice.

## Acknowledgments

The authors gratefully appreciated the team of the Department of Medical Statistics in Guangxi Medical University for their work in the area of statistics in this study.

## Author contributions

**Analyzed and interpreted the data:** DQP, WFW.

**Collected data:** DQP, GXT.

**Conceived and designed the study:** LM, DQP, GXT.

**Conceptualization:** Qinpei Ding, Fangwei Wang, Min Liang.

**Data curation:** Qinpei Ding, Xintong Guo.

**Formal analysis:** Qinpei Ding, Fangwei Wang.

**Review and edit the manuscript:** LM.

**Software:** Qinpei Ding, Fangwei Wang.

**Supervision:** Min Liang.

**Validation:** Qinpei Ding.

**Writing – original draft:** Qinpei Ding.

**Writing – review & editing:** Min Liang.

**Wrote the manuscript:** DQP.
